# Impact of Surface Effects on Support-Loss Mechanisms in Micro-/Nano-Beam Resonators

**DOI:** 10.3390/nano16181175

**Published:** 2026-09-17

**Authors:** Yonglin Chen, Xiaobin Jian, Shuolong Yang, Guangyue Yao, Weijian Jiao, Siyu Chen

**Affiliations:** 1 School of Aerospace Engineering and Applied Mechanics, Tongji University, Shanghai 200092, China; 2 Shanghai Institute of Aircraft Mechanics and Control, Shanghai 200092, China; 3 State Key Laboratory of Flexible Electronics Technology, Tsinghua University, Beijing 100084, China

**Keywords:** MEMS/NEMS resonators, quality factor, support loss, surface effects, elastic wave radiation

## Abstract

The quality factor is a key metric for evaluating the performance of micro-electro-mechanical and nano-electro-mechanical system resonators. Although reducing device dimensions enhances resonator sensitivity, it also intensifies energy dissipation, thereby degrading the quality factor. Among the dominant dissipation mechanisms in micro-/nano-resonators, support loss is strongly influenced by surface effects at small scales, particularly the surface elastic modulus and initial surface stress. In this study, support loss in a double-clamped micro-/nano-beam resonator with surface effects incorporated is investigated. A dynamic model incorporating the surface elastic modulus and initial surface stress is developed based on Euler–Bernoulli beam theory and Gurtin–Murdoch surface elasticity theory. A quality-factor calculation method is then established by combining elastic wave radiation in the supports with an energy-based formulation. The theoretical predictions are validated using a three-dimensional finite element model comprising the resonator with a surface layer, supports, and a perfectly matched layer. Further, the effects of the surface elastic modulus, initial surface stress, characteristic size, and dimensionless geometric parameters on support loss are examined. The results show that the surface elastic modulus and initial surface stress increase support loss and reduce the quality factor, with the initial surface stress exhibiting the stronger influence. The influence of these parameters becomes more pronounced as the characteristic size decreases, and variations in the length-to-thickness and width-to-thickness ratios further modify their contribution to support loss. Mechanistically, surface effects alter the effective bending stiffness and axial force, thereby changing the dynamic loads transmitted to the supports and resulting elastic-wave radiation. These findings provide theoretical insights for the design of high-quality-factor micro-/nano-devices.

## 1. Introduction

Micro-electro-mechanical systems (MEMS) and nano-electro-mechanical systems (NEMS) have integrated micro-/nano-scale mechanical structures, sensing and actuation units, and electronic functional modules on a single platform [1,2]. Owing to their small size, low weight, low power consumption, rapid response, and compatibility with batch fabrication and system integration, MEMS/NEMS devices are widely used in inertial sensing, mass detection, radio-frequency filtering, frequency control, biochemical detection, and precision timing, with applications spanning modern information technology, advanced manufacturing, and intelligent sensing systems [2,3,4,5,6,7,8,9,10].

Among MEMS/NEMS devices, resonators are key dynamic functional units whose vibration characteristics largely determine device performance [2,7,11]. In applications such as frequency selection, signal modulation, weak signal detection, high-precision measurement, and environmental sensing, the natural frequency, mode-shape stability, energy dissipation, and sensitivity to external disturbances directly influence the measurement accuracy, frequency stability, and operational reliability of the system [6,12,13].

With continued device miniaturization and increasing operating frequencies, the demand for micro-/nano-resonators with high stability, high sensitivity, and low power consumption has increased considerably. Consequently, energy dissipation has become a major factor limiting further improvement [7,12,13]. In resonant systems, it governs the vibration decay rate, resonant peak linewidth, frequency resolution, signal-to-noise ratio, and long-term operational stability [12]. The quality factor (*Q*) is widely used to characterize the energy storage capability and dissipation level of a resonator: a higher *Q* indicates lower energy loss per cycle, leading to more stable vibration, high frequency selectivity, improved detection sensitivity, and greater output stability [12]. Consequently, the design and optimization of high-quality-factor resonators and investigation of their dominant dissipation mechanisms have received considerable attention in MEMS/NEMS theory and engineering [7].

Energy dissipation in micro-/nano-resonators arises from multiple coupled mechanisms, including air damping, material internal friction, thermoelastic damping, surface damping, and support loss [7,12,14]. Among these, support loss is a key factor limiting further quality factor improvement [12,14]. Support loss originates from the time-harmonic stress and displacement disturbances generated near the supports during resonator vibration. These disturbances are transmitted through the support into the supporting structure, where they radiate as elastic waves, causing part of the mechanical energy to be continuously dissipated from the localized vibration system and ultimately appearing as irreversible energy dissipation [15,16]. Therefore, under high-vacuum and high-frequency operating conditions, support loss is a critical boundary dissipation mechanism that must be considered when evaluating the upper limit of the resonator quality factor.

Early support-loss models treated energy dissipation as an elastic-wave radiation problem at structural connections. Cross and Lifshitz examined elastic wave transmission at abrupt junctions in thin plates and clarified the relationship between wave transmission characteristics at structural discontinuities and the quality factor, establishing a critical wave-theoretical foundation for subsequent support loss models in micro-/nano-resonators [17]. Hao et al. developed an analytical model for beam-type micro-resonators undergoing in-plane flexural vibration and systematically investigated the effects of geometric and material parameters on the support-loss-limited quality factor [13]. Judge et al. presented a unified analysis of attachment loss in micro-/nanomechanical resonators spanning the limiting cases of thick and thin supports, while Wilson-Rae further interpreted clamping dissipation in nanoelectromechanical resonators from the mesoscopic phonon tunneling perspective, offering further insight into the underlying support loss mechanisms [18,19]. Research later expanded from simple beam structures to disks, shells, and more complex device configurations. Hao and Ayazi extended support-loss analysis to the radial bulk mode of a center-supported disk resonator and obtained an analytical expression for the support-loss-limited quality factor [20]. Hao and Xu further analyzed the displacement response of supports subjected to time-harmonic loading, revealing the displacement field associated with elastic-wave propagation from the resonator into the supports and providing a clearer mechanical interpretation of the radiation mechanism [16]. These studies demonstrate the evolution of support-loss analysis from simplified beam models to the prediction and evaluation of quality factors in devices with increasingly complex geometries, boundary conditions, and multiphysics constraints.

In parallel with mechanistic studies, strategies for suppressing support loss have been investigated. Ma et al. theoretically investigated the effects of axial pretension and geometric nonlinearity, demonstrating that pretension reduces thermoelastic damping but may increase support loss. They further showed that conventional linear support-loss models become inadequate under large-deflection conditions, making geometric nonlinear corrections necessary [21,22]. Xiao et al. introduced a two-dimensional (2D) honeycomb photonic crystal into a piezoelectric MEMS resonator and verified through finite element analysis and experiments that it effectively suppresses support loss, increasing the average quality factor by approximately 1.7 times. These findings demonstrate that wave propagation regulation using periodic structures is critical for support-loss optimization [23].

The aforementioned studies establish a systematic foundation for the mechanistic modeling and suppression-oriented design of support loss. However, as MEMS/NEMS devices continue to evolve toward smaller dimensions, higher operating frequencies, and greater sensitivity, conventional continuum-based support-loss models must be extended to account for size-induced surface effects.

**Figure 1:**
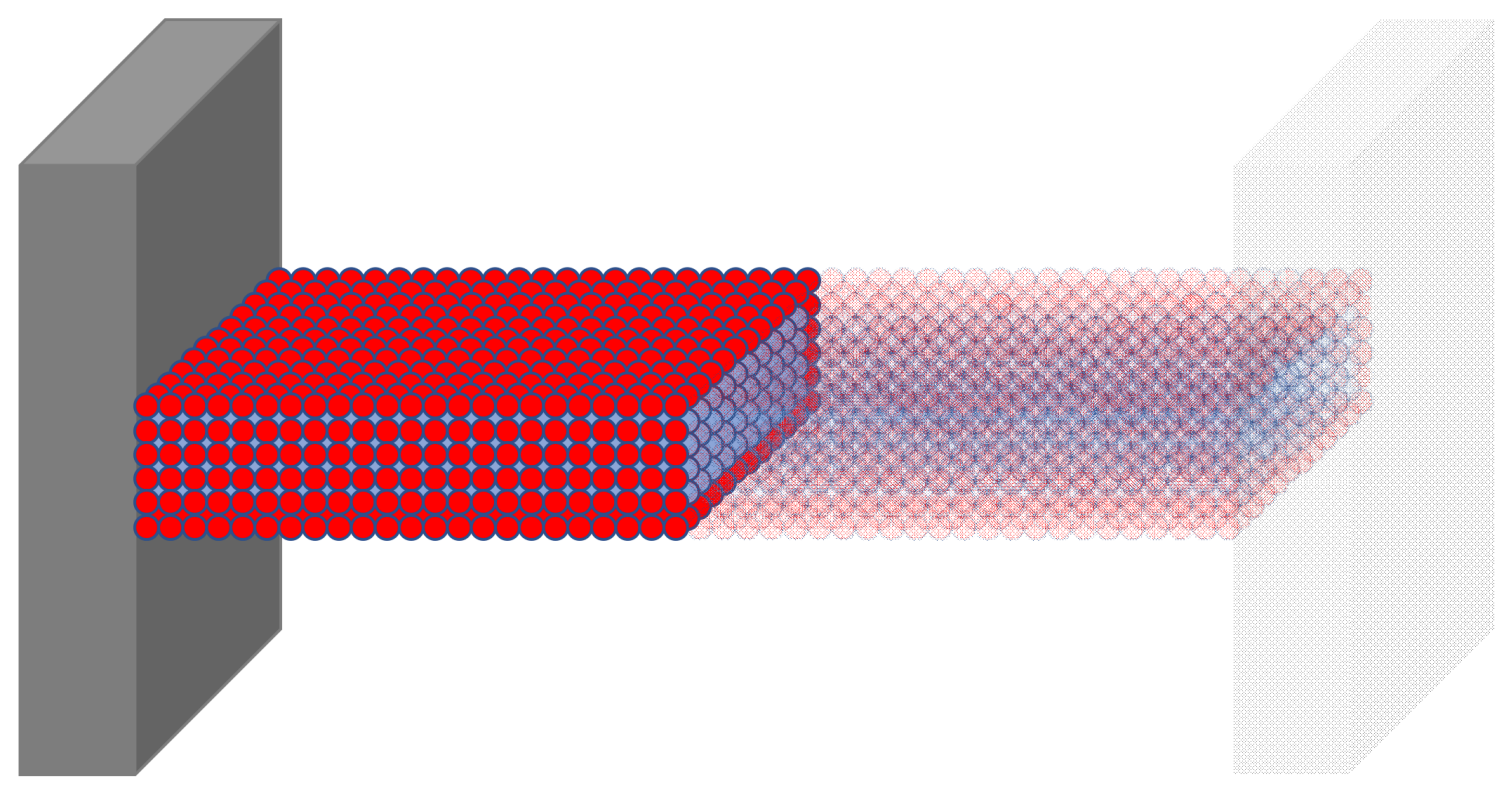
Schematic of the molecular composition of a micro-/nanoscale resonator (the red region indicates the surface-layer molecules).

When the characteristic dimensions of a structure decrease to the micro-/nanoscale, its surface-area-to-volume ratio increases rapidly, while the coordination environment, bonding state, and energy distribution of surface molecules differ considerably from those of bulk molecules, resulting in pronounced size-dependent mechanical behavior [24,25]. For typical micro-/nano-structures such as beams, nanowires, and thin films, conventional continuum theory treats the material as a homogeneous bulk body and neglects the independent mechanical properties of the surface. Although this assumption is generally valid at the macroscale, its applicability diminishes considerably at the micro-/nanoscale. To account for these surface-induced size effects, Gurtin and Murdoch developed surface elasticity theory, wherein the free surface is modeled as a zero-thickness elastic layer attached to the bulk material and governed by an independent constitutive relationship. Consequently, the overall mechanical response is modified by the initial surface stress and surface elastic modulus [26]. Figure 1 illustrates the molecular structure of a micro-/nanoscale resonator, highlighting the microscopic origin of surface effects. Subsequent studies further showed that the size-dependent behavior of micro-/nano-structures is governed by the competition between surface elasticity and bulk elasticity and can be characterized by an intrinsic length scale determined jointly by surface elastic parameters and bulk elastic properties [27]. Hence, treating the surface layer as an independent mechanical domain rather than merely a geometric boundary has become a fundamental theoretical approach for capturing size effects.

Investigations on the influence of surface effects on the mechanical behavior of micro-/nano-structures have been conducted through experimental observations and theoretical modeling. Jing et al. measured the apparent elastic modulus of silver nanowires with different diameters using contact atomic force microscopy and showed that the effective Young’s modulus deviates considerably from the macroscopic material constant as the size changes, confirming the influence of surface effects on the elastic response of nano-structures [28]. Cuenot et al. further found that the apparent elastic modulus of silver and lead nanowires and polypyrrole nanotubes increases as the diameter decreases, attributing this behavior to surface tension effects [29]. Chen et al. demonstrated through bending tests on ZnO nanowires that the Young’s modulus increases considerably when the diameter falls below approximately 120 nm, revealing pronounced surface stiffening [30]. Roy et al. combined laser Doppler vibrometry with two-point bending experiments and showed that the fracture strain and strength of ZnO nanowires are highly sensitive to surface defects, indicating that the surface state critically affects their mechanical reliability [31]. Guo and Zhao introduced surface effects into the bending analysis of nanobeams and showed that the surface layer contribution to bending stiffness increases rapidly as the transverse dimension decreases, leading to considerable size dependence [32]. He and Lilley further investigated the effects of surface stress and surface elasticity on the static bending of nanowires based on the Young-Laplace equation, demonstrating that surface effects can either stiffen or soften the structure depending on the boundary conditions, cross-sectional dimensions, and surface parameter values [33]. These studies demonstrate that surface effects are not merely small corrections to material constants but can fundamentally alter the effective stiffness and deformation behavior of micro-/nano-structures.

Dynamic studies have further clarified the influence of surface effects on natural frequencies and resonance characteristics [34]. Park investigated the resonance of silicon nanowires and showed that considerable coupling exists between surface stress and boundary constraints. For double-clamped structures, the equivalent axial stress induced by initial surface stress considerably alters the resonant frequency, while cantilever structures exhibit different responses [35]. Yao and Chen further investigated the resonance characteristics of nanowires under different boundary conditions and showed that the influence of surface effects on resonant frequency becomes more considerable as the characteristic dimension decreases and slenderness ratio increases. Additionally, they found that double-clamped and cantilever beams exhibit different sensitivities to surface effects [36]. These studies show that surface effects influence the static stiffness of micro-/nano-structures, including their vibration and resonance characteristics, by modifying the effective bending stiffness, initial stress state, and inertia stiffness balance.

In summary, previous studies on support loss in micro-/nano-resonators have clarified the roles of structural configuration, support geometry, boundary conditions, and related factors in determining the quality factor, while studies of surface effects have provided insights into effective stiffness, natural frequency, and size-dependent behavior in micro-/nano-structures. However, the impact of surface effects on support loss remains poorly understood. Existing support loss models are often based on conventional continuum theory and generally neglect the surface elastic modulus and initial surface stress. For double-clamped micro-/nano-beams, a systematic understanding of how surface effects modify the local mechanical response near the support region, thereby influencing elastic-wave radiation into the support, is still lacking.

In this study, support loss in a double-clamped micro-/nano-beam resonator is investigated by accounting for surface effects. A theoretical model incorporating the surface elastic modulus and initial surface stress is developed based on Euler–Bernoulli beam theory and Gurtin–Murdoch surface elasticity theory, while the corresponding quality factor is obtained by combining elastic-wave radiation into the supports with an energy-based method. A three-dimensional (3D) finite element model comprising the resonator with a surface layer, supports, and a perfectly matched layer (PML) is then established to validate the theoretical predictions. The effects of surface parameters and dimensionless geometric parameters on support loss and the quality factor are systematically analyzed, providing theoretical insight for the design and optimization of high-quality-factor micro-/nano-resonators.

## 2. Theoretical Model

### 2.1. Structural Model and Basic Assumptions

**Figure 2:**
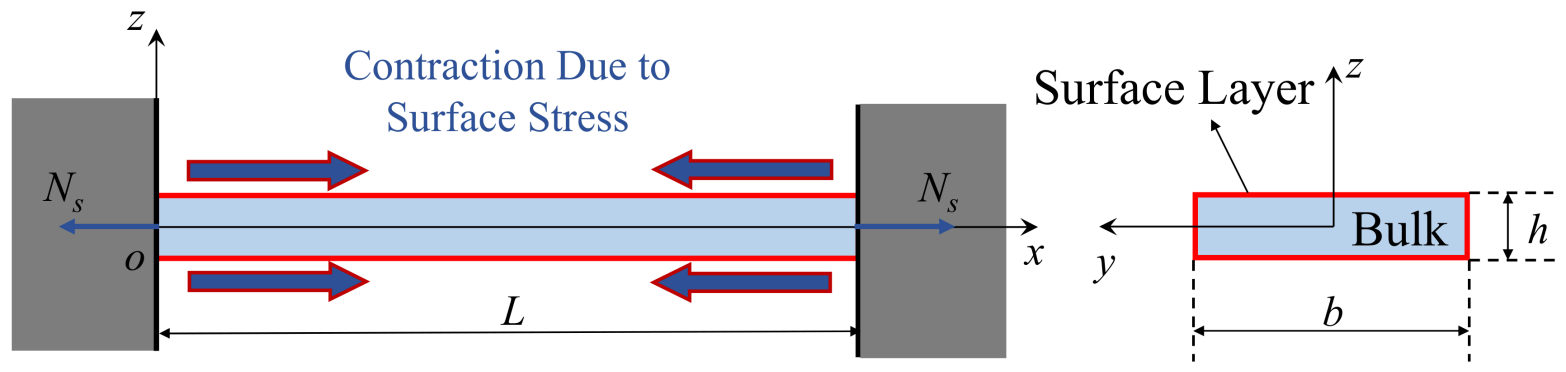
Schematic of the overall configuration and cross-section of a double-clamped micro-/nano-beam resonator considering surface effects (dark blue arrows: contraction tendency of the surface layer induced by surface stress; thin blue arrows: equivalent axial tensile force ($N_{s}$) resulting from clamped boundary constraints).

As shown in Figure 2, a double-clamped micro-/nano-beam resonator with a rectangular cross-section is considered. The dynamic response and dissipation behavior of vibrating beams depend strongly on the specific support and boundary constraints [37], and this work focuses on the double-clamped configuration to investigate how surface effects modulate the clamping interface loads and the resulting elastic-wave radiation. A Cartesian coordinate system is adopted, with the beam axis along the *x*-direction, cross-sectional width along the *y*-direction, and height along the *z*-direction. The beam has length *L*, width *b*, and height *h*, and its spatial domain is expressed as follows: 0≤x≤L, −0.5b≤y≤0.5b, and −0.5h≤z≤0.5h. Both resonator ends are connected to semi-infinite supports. The left panel of Figure 2 shows the overall beam-support configuration, while the right panel shows the rectangular cross-section and surface layer. The gray region indicates the support, blue indicates the resonator bulk material, and red outlines the surface layer, which represents the independent mechanical properties of the free surface at the micro-/nanoscale. The surface layer is subjected to an initial surface stress $\tau_{s}$ (N/m), representing the stress arising from the imbalance of intermolecular forces within the surface layer [26]. In the present model, only the tensile scenario ($\tau_{s}>0$) is analyzed. This treatment aligns with the physical reality of practical silicon micro-/nano-resonators, where the constraint of clamped boundaries on surface contraction typically induces a tensile surface stress. Furthermore, a compressive surface stress ($\tau_{s}<0$) would cause beam buckling, rendering the present linear theoretical model invalid. Regarding the clamped ends, because they are bonded interfaces rather than free surfaces, they carry no surface layer; therefore, the initial surface stress enters the governing model solely through the equivalent axial prestress on the free surfaces.

To account for the influence of surface effects on the dynamic behavior of the resonator at the micro-/nano-scale, this study adopts the Gurtin–Murdoch surface elasticity theory, wherein the free surface is modeled as a zero-thickness elastic layer bonded to the bulk material and possessing independent mechanical properties [26,33,38]. Within this framework, the surface layer has a constitutive relationship distinct from that of the bulk. Its effects are governed by two surface parameters: the surface elastic modulus (modifies the effective stiffness of the structure) and initial surface stress (acts as an additional axial prestress term in the dynamic equation of the double-clamped beam) [27,35,36]. These surface parameters alter the effective bending stiffness, natural frequency, and internal force distribution, thereby modifying the boundary mechanical response at the supports and leading to support loss.

Euler–Bernoulli beam theory is adopted to describe the transverse flexural vibration of the resonator. Additionally, the beam cross-section remains plane and perpendicular to the neutral axis during deformation, while transverse shear deformation and rotary inertia are neglected [15,27,33]. We hereby assume that the resonator and its support structure have the same dimension in the *y* direction and the resonator undergoes small-amplitude flexural vibration in the *xz*-plane, so that the support loss in the resonator can be analyzed using a two-dimensional model. To isolate support loss as the dissipation mechanism, the following assumptions are made: (1) the resonator undergoes linear, small-amplitude vibration in low-order modes; (2) the support material satisfies a linear elastic constitutive relation; (3) the support is approximated as a semi-infinite elastic body to characterize elastic-wave radiation toward the far field; (4) only the first-order flexural vibration response of the resonator in the xz-plane and corresponding support loss are considered. These assumptions are consistent with those commonly adopted in analytical support loss models, support wave-response analyses, and related reviews.

Under these assumptions, surface effects influence support loss through two primary mechanisms. First, the surface elastic modulus and initial surface stress modify the effective stiffness and vibration characteristics of the resonator. Second, they alter the shear force, bending moment, and displacement-gradient distributions near the supports, thereby changing the excitation of elastic waves and associated energy efficiency. The resulting variation in radiated energy per cycle is reflected in the support-loss-limited quality factor and forms the basis of the theoretical and finite element analyses presented below.

### 2.2. Dynamic Equation of the Double-Clamped Beam Considering Surface Effects

When surface effects are considered, the governing equation of the double-clamped beam is expressed as follows [26]:

(1) $$\rho A\frac{\partial^{2}w\left(x,t\right)}{\partial t^{2}}+\left(EI_{eff}\right)\frac{\partial^{4}w\left(x,t\right)}{\partial x^{4}}-N_{s}\frac{\partial^{2}w\left(x,t\right)}{\partial x^{2}}=0,$$

where ρ denotes the resonator density, A=bh is the cross-sectional area, $w\left(x,t\right)$ is the transverse displacement of the beam, $EI_{eff}$ is the equivalent bending stiffness considering the surface elastic modulus, and $N_{s}$ is the equivalent axial prestress induced by the initial surface stress.

According to Gurtin–Murdoch surface elasticity theory [26], the equivalent bending stiffness of a beam with a rectangular cross-section is expressed as follows:

(2) $$EI_{eff}=\frac{E_{b}bh^{3}}{12}+\frac{E_{s}bh^{2}}{2}+\frac{E_{s}h^{3}}{6},$$

where $E_{b}$ is the elastic modulus of the bulk material, and $E_{s}$ is the surface elastic modulus. The first term represents the bulk material contribution, whereas the second and third terms represent the contributions of the upper and lower surfaces and the side surfaces, respectively.

If all free surfaces have the same initial tensile surface stress ($\tau_{s}$), integration along the cross-sectional perimeter yields the equivalent axial prestress:

(3) $$N_{s}=2\tau_{s}\left(b+h\right).$$

### 2.3. Modal Response of the Beam and Interface Load

If the beam is assumed to undergo harmonic vibration, the transverse displacement is expressed as

(4) $$w\left(x,t\right)=\phi \left(x\right)e^{i\omega t},$$

where $\phi \left(x\right)$ is the mode shape function, ω is the angular frequency, and $i=\sqrt{-1}$.

Substituting Equation (4) into Equation (1) yields the governing ordinary differential equation for the mode shape function, $\phi \left(x\right)$.

(5) $$EI_{eff}\phi^{\left(4\right)}\left(x\right)-N_{s}\phi^{\prime \prime }\left(x\right)-\rho A\omega^{2}\phi \left(x\right)=0.$$

If $\phi \left(x\right)=e^{\lambda x}$ is assumed, the corresponding characteristic equation becomes

(6) $$EI_{eff}\lambda^{4}-N_{s}\lambda^{2}-\rho A\omega^{2}=0.$$

Letting $r=\lambda^{2}$ yields

(7) $$EI_{eff}r^{2}-N_{s}r-\rho A\omega^{2}=0.$$

Solving this equation gives two sets of characteristic roots, and the corresponding wavenumber parameters can be written as

(8) $$r=\frac{N_{s}\pm \sqrt{N_{s}^{2}+4EI_{eff}\rho A\omega^{2}}}{2EI_{eff}}.$$

For the silicon-based double-clamped micro-/nano-beam considered in this study, if all free surfaces undergo tensile initial surface stress, $N_{s}>0$, the following relationships are obtained:

(9) $$\lambda_{1}=\sqrt{\frac{N_{s}+\sqrt{N_{s}^{2}+4EI_{eff}\rho A\omega^{2}}}{2EI_{eff}}};$$

(10) $$\lambda_{2}=\sqrt{\frac{-N_{s}+\sqrt{N_{s}^{2}+4EI_{eff}\rho A\omega^{2}}}{2EI_{eff}}}.$$

The general form of the mode shape function is

(11) $$\phi \left(x\right)=C_{1}\cosh\left(\lambda_{1}x\right)+C_{2}\sinh\left(\lambda_{1}x\right)+C_{3}\cos\;\left(\lambda_{2}x\right)+C_{4}\sin\;\left(\lambda_{2}x\right).$$

For a double-clamped beam, the boundary conditions are

(12) $$\phi \left(0\right)=0;$$

(13) $$\phi^{\prime }\left(0\right)=0;$$

(14) $$\phi \left(L\right)=0;$$

(15) $$\phi^{\prime }\left(L\right)=0.$$

Substituting Equations (12)–(15) into Equation (11) results in

(16) $$C_{1}+C_{3}=0;$$

(17) $$C_{2}\lambda_{1}+C_{4}\lambda_{2}=0;$$

(18) $$C_{1}\cosh\left(\lambda_{1}L\right)+C_{2}\sinh\left(\lambda_{1}L\right)+C_{3}\cos\;\left(\lambda_{2}L\right)+C_{4}\sin\;\left(\lambda_{2}L\right)=0;$$

(19) $$C_{1}\lambda_{1}\sinh\left(\lambda_{1}L\right)+C_{2}\lambda_{1}\cosh\left(\lambda_{1}L\right)-C_{3}\lambda_{2}\sin\;\left(\lambda_{2}L\right)+C_{4}\lambda_{2}\cos\;\left(\lambda_{2}L\right)=0.$$

Solving Equations (16)–(19) yields

(20) $$2\lambda_{1}\lambda_{2}\left[1-\cosh\left(\lambda_{1}L\right)\cos\;\left(\lambda_{2}L\right)\right]-\left(\lambda_{2}^{2}-\lambda_{1}^{2}\right)\sinh\left(\lambda_{1}L\right)\sin\;\left(\lambda_{2}L\right)=0;$$

(21) $$C_{1}:C_{2}:C_{3}:C_{4}=1:-\frac{\cosh\left(\lambda_{1}L\right)-\cos\;\left(\lambda_{2}L\right)}{\sinh\left(\lambda_{1}L\right)-\frac{\lambda_{1}}{\lambda_{2}}\sin\;\left(\lambda_{2}L\right)}:-1:\frac{\lambda_{1}}{\lambda_{2}}\frac{\cosh\left(\lambda_{1}L\right)-\cos\;\left(\lambda_{2}L\right)}{\sinh\left(\lambda_{1}L\right)-\frac{\lambda_{1}}{\lambda_{2}}\sin\;\left(\lambda_{2}L\right)}.$$

The corresponding mode shape function, $\phi \left(x\right)$, is written as

(22) $$\phi \left(x\right)=U\left\{\cosh\left(\lambda_{1}x\right)-\cos\;\left(\lambda_{2}x\right)-\chi \left[\sinh\left(\lambda_{1}x\right)-\frac{\lambda_{1}}{\lambda_{2}}\sin\;\left(\lambda_{2}x\right)\right]\right\},$$

where U is the normalized amplitude, and χ is the shape coefficient determined based on the boundary conditions:

(23) $$\chi =\frac{\cosh\left(\lambda_{1}L\right)-\cos\;\left(\lambda_{2}L\right)}{\sinh\left(\lambda_{1}L\right)-\frac{\lambda_{1}}{\lambda_{2}}\sin\;\left(\lambda_{2}L\right)}.$$

Accurate characterization of the interface loads transmitted from the resonator to the support is critical for support-loss analysis. Among these loads, the shear-force-induced energy dissipation is the dominant contributor to support loss [39]. The contribution of the bending moment to the support loss is neglected in the present study, as it has been demonstrated by Chen et al. [39] that moment-induced energy dissipation at the micro-/nano-scale can be neglected compared with that caused by the transverse shear force.

According to Euler–Bernoulli beam theory, the transverse shear force at the clamped end of the resonator is obtained from the third derivative of the mode shape function

(24) $$F_{s}=-{\left.EI_{eff}\frac{\partial^{3}w}{\partial x^{3}}\right|}_{x=0}-{\left.N_{s}\frac{\partial w}{\partial x}\right|}_{x=0}=U\chi \lambda_{1}\sqrt{N_{s}^{2}+4EI_{eff}\rho A\omega^{2}}e^{i\omega t}=F_{s0}e^{i\omega t}.$$

**Figure 3:**
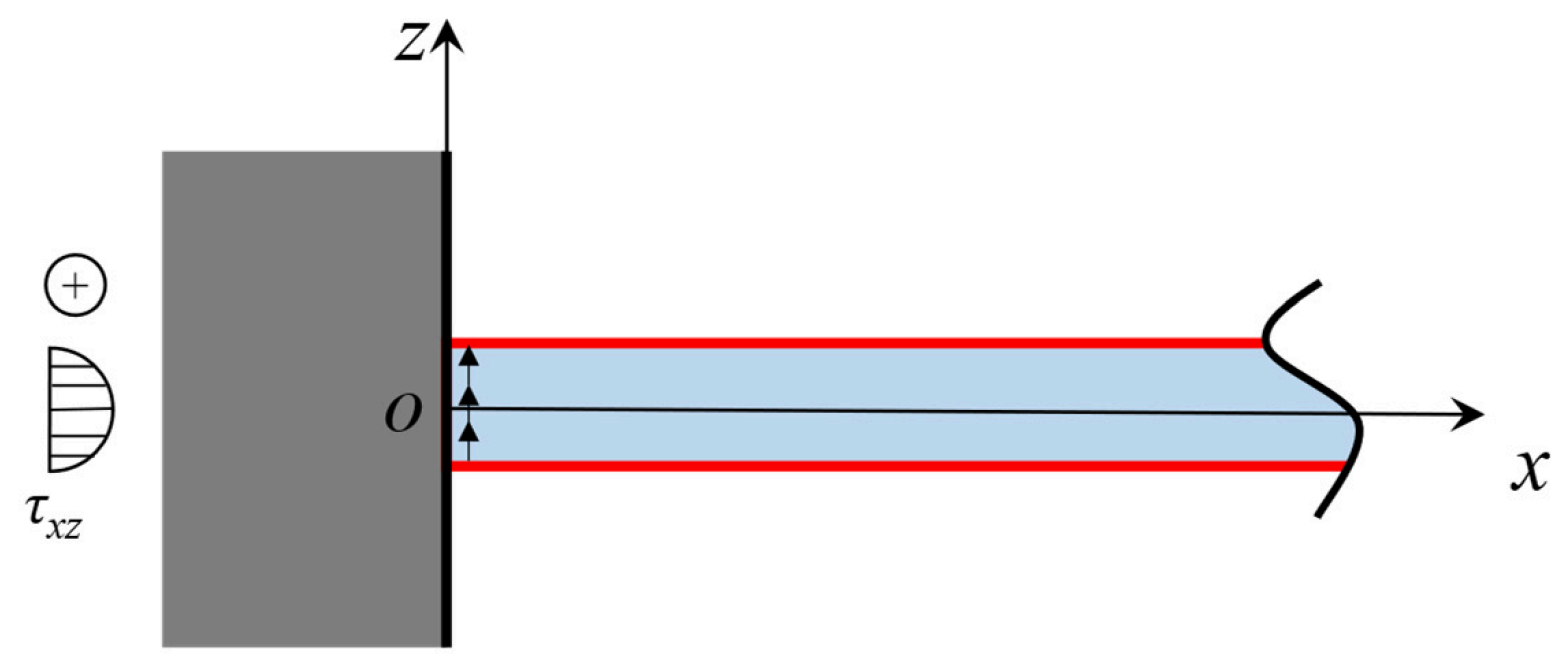
Schematic of the stress distribution at the resonator-substrate interface (blue region: bulk material of the beam; red region: surface layer with surface effects; gray region: support; the parabolic profile on the left illustrates the variation of shear stress along the thickness direction (*z*-axis) at the interface).

The shear stress, $\tau \left(z\right)$, in the connection region between the resonator and support can be approximated from the clamped-end shear force ($F_{s}$) and the shear stress distribution of a rectangular cross-section, as shown in Figure 3. The corresponding time-harmonic shear stress is

(25) $$\tau \left(z\right)=\frac{F_{s}S^{*}\left(z\right)}{bI},$$

where

(26) $$S^{*}\left(z\right)=\frac{b}{2}\left(\frac{h^{2}}{4}-z^{2}\right).$$

Substituting Equation (24) into Equation (25) gives

(27) $$\tau \left(z\right)=F_{\tau }e^{i\omega t}=\left\{\begin{array}{ll} \tau_{0}\left(\frac{h^{2}}{4}-z^{2}\right)e^{i\omega t} & \mathrm{for}\;\left|z\right|\leq h/2\\ 0 & \mathrm{for}\;\left|z\right|>h/2 \end{array}\right.,$$

where

(28) $$\tau_{0}=\frac{1}{2I}U\chi \lambda_{1}\sqrt{N_{s}^{2}+4EI_{eff}\rho A\omega^{2}}.$$

### 2.4. Elastic Wave Radiation and Support-Loss-Related Quality Factor

The quality factor (*Q*) is expressed as follows:

(29) $$Q=2\pi \frac{W}{\Delta W},$$

where W denotes the maximum vibration energy stored in the resonator, and ΔW denotes the energy dissipated during each vibration cycle. W is independent of the support form because it corresponds to the strain energy of each resonator vibration mode.

(30) $$W=\frac{1}{2}\rho A\omega^{2}\int_{0}^{L}{\left|\phi \left(x\right)\right|}^{2}dx=\frac{1}{2}\rho AL\omega^{2}U^{2}.$$

The support loss (ΔW) in each vibration cycle is expressed as the power integral of stress and velocity at the support interface [39]

(31) $$\Delta W=\int_{0}^{2\pi /\omega }\int_{\mathrm{Attachment}\;\mathrm{region}}\sigma_{ij}\left(t\right)\cdot u_{j}\left(t\right)\;dsdt.$$

The displacement response of a semi-infinite elastic support subjected to time-harmonic shear stress is expressed as follows [39]:

(32) $${\left.v\left(z\right)\right|}_{x=0}=\frac{2\tau_{0}\omega^{2}}{\rho \pi }\left\{\int_{0}^{\infty }\frac{\cos\;\left(\xi z\right)\left[2\sin\;\left(\frac{h\xi }{2}\right)-b\xi \cos\;\left(\frac{h\xi }{2}\right)\right]\sqrt{\xi^{2}-\frac{\omega^{2}}{c_{T}^{2}}}}{\xi^{3}{\left(\omega^{2}-2c_{T}^{2}\xi^{2}\right)}^{2}-4c_{T}^{4}\xi^{2}\sqrt{\xi^{2}-\frac{\omega^{2}}{c_{T}^{2}}}\sqrt{\xi^{2}-\frac{\omega^{2}}{c_{L}^{2}}}}d\xi \right\}e^{i\omega t},$$

where ξ is the Fourier transform parameter, and $c_{L}$ and $c_{T}$ are the longitudinal and transverse wave velocities in the support medium under the plane-stress assumption, respectively.

(33) $$c_{L}^{2}=\frac{E}{\rho \left(1-\mu^{2}\right)},$$

(34) $$c_{T}^{2}=\frac{E}{2\rho \left(1+\mu \right)}.$$

Substituting Equations (27) and (32) into the power integral in Equation (31) gives

(35) ΔW=∫02πω∫−h2h2ReFτ⋅eiωt⋅Re∂v∂tdsdt =−2πbω2τ02ρπ∫−h2h2(h24−z2)Im∫0maxωcT,ωcLcos ξz2sinhξ2−bξcos hξ2ξ2−ω2cT2ξ3ω2−2cT2ξ22−4cT4ξ2ξ2−ω2cT2ξ2−ω2cL2dξdz.

## 3. Finite Element Model and Computational Method

### 3.1. Establishing the 3D Finite Element Model (FEM)

**Figure 4:**
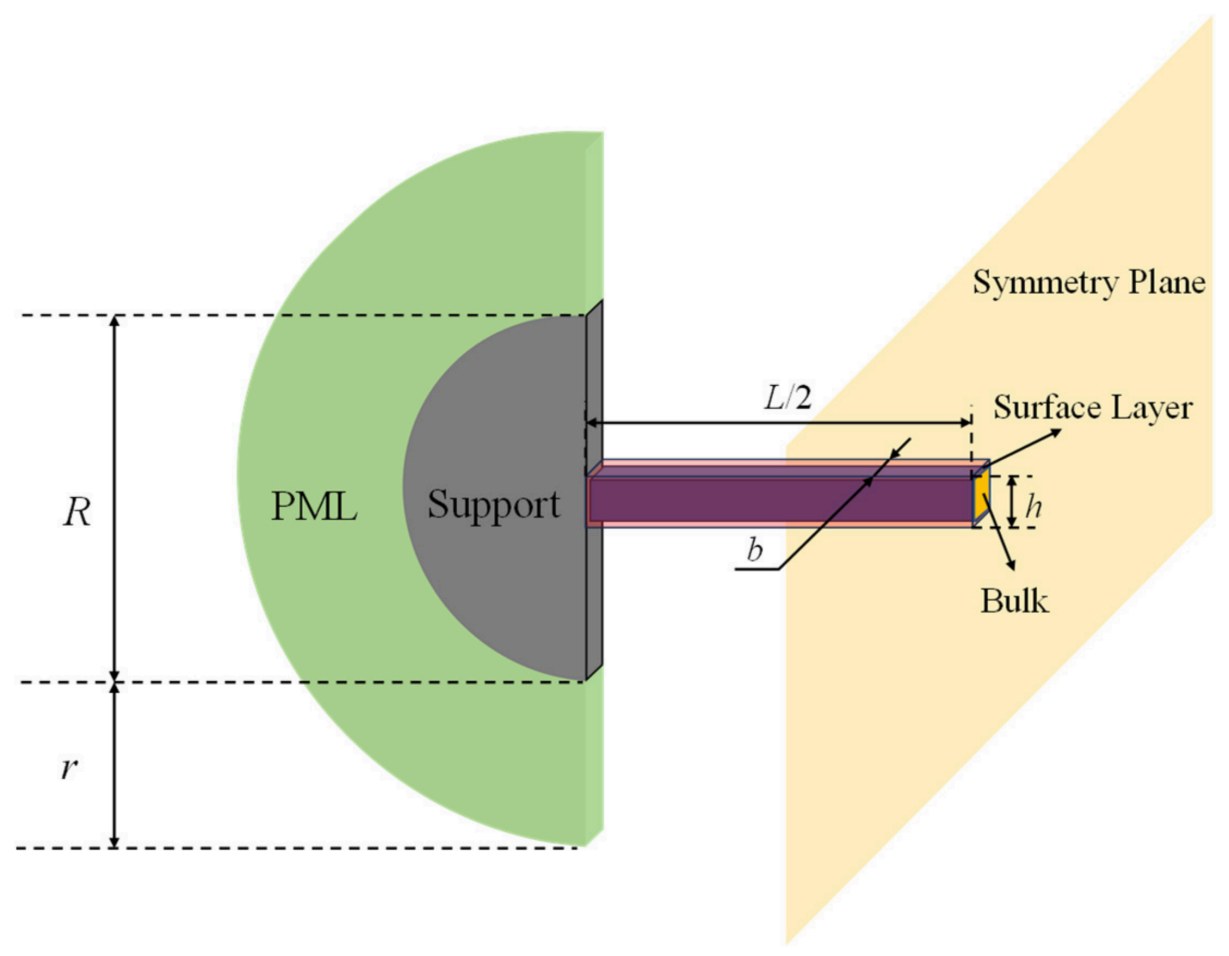
Three-dimensional symmetric half-model for the finite element analysis.

To validate the theoretical model and further investigate the influence of surface effects on support loss in double-clamped micro-/nano-beam resonators, a 3D finite element model is developed in COMSOL Multiphysics 6.2. As shown in Figure 4, the model comprises the resonator, an equivalent surface layer, the supports, and a PML. The PML simulates an unbounded domain through a non-reflective absorbing boundary, thereby minimizing truncation errors. The blue region represents the resonator bulk material, red the equivalent surface layer, gray the elastic supports, and green the PML. Given the structural and modal symmetry, only one half of the double-clamped beam is retained in the finite element model. Symmetry boundary conditions are applied on the symmetry plane to reduce computational cost while maintaining solution accuracy. The yellow surface indicates the symmetry plane of the half model, which corresponds to the truncation plane of the computational domain.

The resonator is a double-clamped micro-/nano-beam with a rectangular cross-section, with one end connected to the support and the other corresponding to the symmetry plane. To represent the elastic-wave radiation from the support region into the surrounding medium, a semi-cylindrical support having the same thickness as the beam, enclosed by a matching semi-cylindrical PML, is adopted. This configuration allows waves generated at the support region to propagate radially through the support and to be gradually attenuated in the outer absorbing region, thereby reducing reflections from the finite computational boundary [40]. Compared with a simple block-type support model, the semi-cylindrical configuration better captures the geometric spreading of elastic waves toward the far field in support-loss analysis.

To balance computational accuracy and efficiency, the support and PML dimensions are selected based on the characteristic wavelength. The support diameter and PML thickness are expressed as $R=\frac{2c_{L}}{\omega }+h$ and $r=\frac{c_{L}}{\omega }$, respectively. These dimensions provide sufficient propagation distance for elastic waves before entering the PML while ensuring effective absorption within the absorbing region [40,41]. A fixed constraint is applied at the outer boundary of the PML to prevent rigid-body motion and improve eigenfrequency stability. This constraint does not represent a physical boundary; rather, it is a numerical treatment used together with the PML to attenuate outgoing waves and prevent reflections from the truncated computational domain.

### 3.2. Implementation of Surface Effects Modeling

The theoretical analysis is based on Gurtin–Murdoch surface elasticity theory, wherein the free surfaces of the resonator are modeled as zero-thickness layers with independent mechanical properties characterized by the surface elastic modulus and initial surface stress. To incorporate these effects into the three-dimensional finite element model, an ultrathin surface layer is introduced on the free surfaces of the resonator and assigned equivalent material properties consistent with the theoretical surface formulation.

The surface elastic parameters are converted into equivalent bulk material properties of the thin layer to capture the additional contribution of surface elasticity to bending stiffness in the finite element model. The initial surface stress is introduced as an equivalent in-plane prestress field within the surface layer. Hence, the finite element model accounts for the surface-induced modifications of the effective stiffness and local stress state of the resonator. The equivalent surface layer uniformly covers the free surfaces of the resonator with a thickness of *t_s_* = 0.23 nm, corresponding to the characteristic thickness of a silicon monolayer. The surface layer is implemented using shell elements. Accordingly, the equivalent bulk properties of the layer are obtained by smearing the surface parameters over this thickness: the equivalent Young’s modulus is $E_{s}/t_{s}$, and the equivalent in-plane prestress is $\tau_{s}/t_{s}$. Except at the extreme nanoscale, this thickness is negligible compared with the resonator dimensions and therefore does not considerably affect the overall geometry, while preserving the influence of surface effects on the local mechanical response.

This approach is adopted for two main reasons: (1) when the surface layer thickness is at the atomic scale and smaller than the cross-sectional dimensions of the resonator, its contribution to the overall mass and bulk stiffness remains limited, thereby minimizing additional numerical errors [42]; (2) the equivalent surface layer effectively captures the independent mechanical behavior of free surfaces at the micro-/nanoscale and enables the theoretical surface to be incorporated consistently into the finite element model [43,44]. Therefore, the equivalent thin surface layer method provides a balance between physical accuracy and computational efficiency and is suitable for investigating the coupling between surface effects and support loss.

### 3.3. Extraction Method for the Support-Loss-Limited Quality Factor and Convergence Analysis

In eigenfrequency analysis, elastic wave radiation through the support region introduces a complex eigenfrequency [41]. The real part corresponds to the resonant frequency, while the imaginary part represents attenuation associated with energy dissipation. Hence, the support-loss-limited quality factor can be extracted directly from the complex eigenfrequency without introducing an artificial damping model, thereby enabling a more direct evaluation of support loss.

The complex eigenfrequency is denoted as *f*; hence, the support-loss-limited quality factor is expressed as

(36) Q=Ref2Imf,

where Ref and Imf denote the real and imaginary parts of the complex eigenfrequency, respectively. This approach simultaneously provides the resonant frequency and dissipation characteristics within a unified eigenvalue framework, making it suitable for support-loss analysis dominated by elastic-wave radiation.

**Figure 5:**
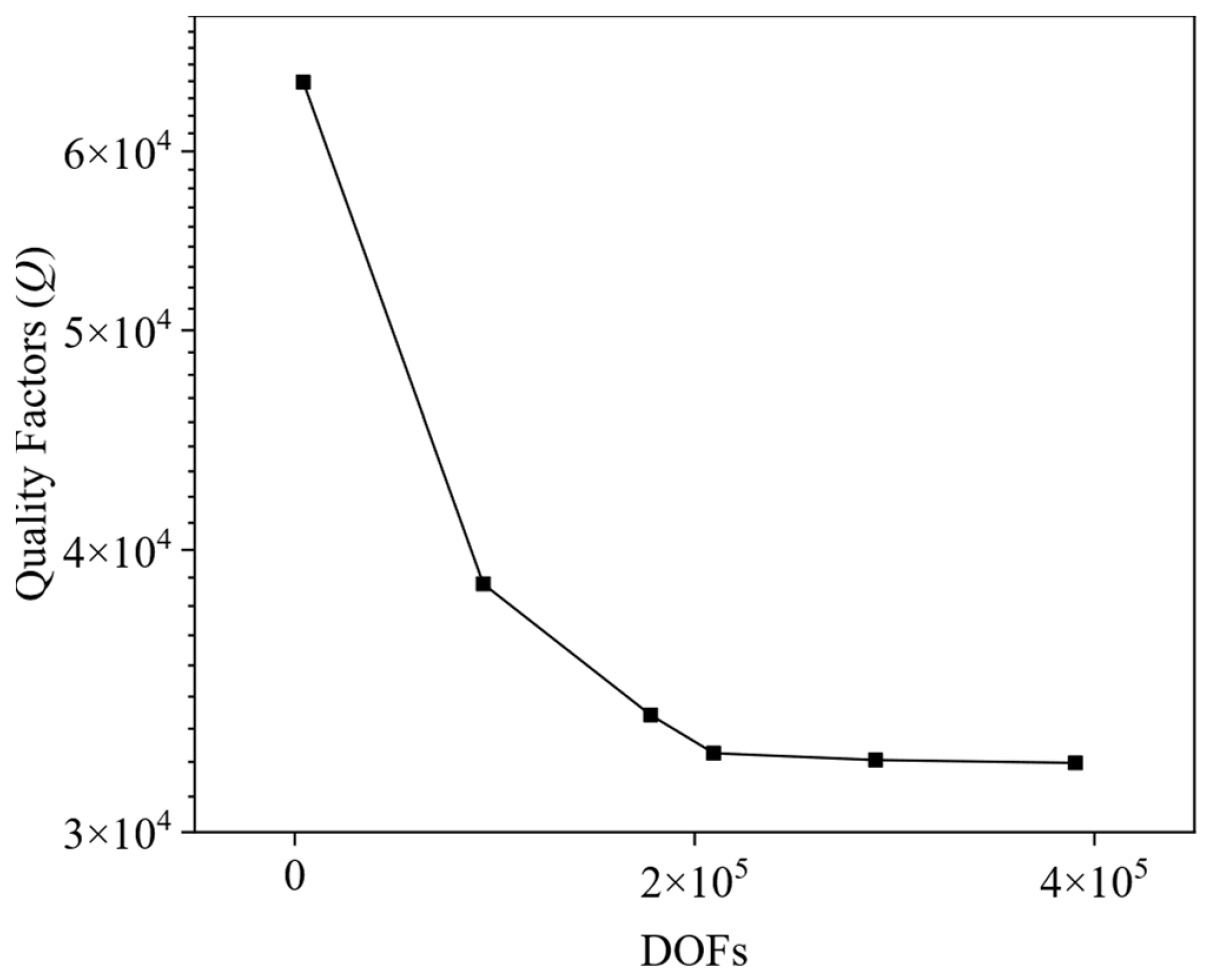
Mesh convergence analysis results.

A mesh convergence analysis is performed to verify the accuracy of the numerical results. Because support loss is highly sensitive to stress concentration near the supports, displacement gradients, and elastic-wave propagation accuracy, local mesh refinement is applied to the resonator, beam-support interface, and surrounding support region [45]. A gradually coarsened mesh is adopted in the far-field support and PML regions to balance wave absorption accuracy and computational efficiency. For each mesh density, the complex eigenfrequency and corresponding quality factor are computed, while the variations between successive mesh levels are evaluated. Figure 5 shows the convergence curve of the quality factor as a function of the number of degrees of freedom (DOFs). The quality factor gradually stabilizes as the mesh is refined. When the DOFs become sufficiently large, further refinement results in only slight variations, indicating that the numerical solution has achieved mesh-independent convergence.

## 4. Results and Discussion

Based on the surface-effect-incorporated support loss model described in Section 2 and the 3D finite element model presented in Section 3, the influence of surface parameters, characteristic dimensions, and dimensionless geometric parameters on support loss and the quality factor of double-clamped micro-/nano-resonators is investigated. The theoretical predictions are validated against the finite element results.

The effects of the surface elastic modulus ($E_{s}$) and initial surface stress ($\tau_{s}$) on support loss are discussed in Section 4.1 and Section 4.2, respectively. With dimensionless geometric parameters L/h and b/h held constant, the size-dependent influence of surface effects is investigated by varying the characteristic dimension, h, as presented in Section 4.3. Finally, the effects of L/h and b/h on the support loss and degree of surface-effect modulation are evaluated (Section 4.4).

For consistency in the parametric analyses, all parameters except the one under investigation are fixed at their default values. The default values and variation ranges are listed in Table 1. The silicon resonator, the equivalent surface layer, and the supports are all treated as homogeneous, isotropic, linear elastic materials.

For consistency, $Q_{0}$ denotes the quality factor obtained without surface effects and $Q_{s}$ represents that obtained when surface effects are included. In the parameter ranges considered, $Q_{s}$ is smaller than $Q_{0}$ because of the influence of surface effects. The relative variation, $Q_{e}=\frac{Q_{0}-Q_{s}}{Q_{0}}\times 100\%$, is introduced as a normalized measure of the influence of surface effects on support loss and quality factor, with larger $Q_{e}$ indicating a stronger surface-effect contribution. Both theoretical and finite element results are presented to validate model consistency and clarify the mechanisms governing how different parameters influence support loss.

### 4.1. Effect of Surface Elastic Modulus

**Figure 6:**
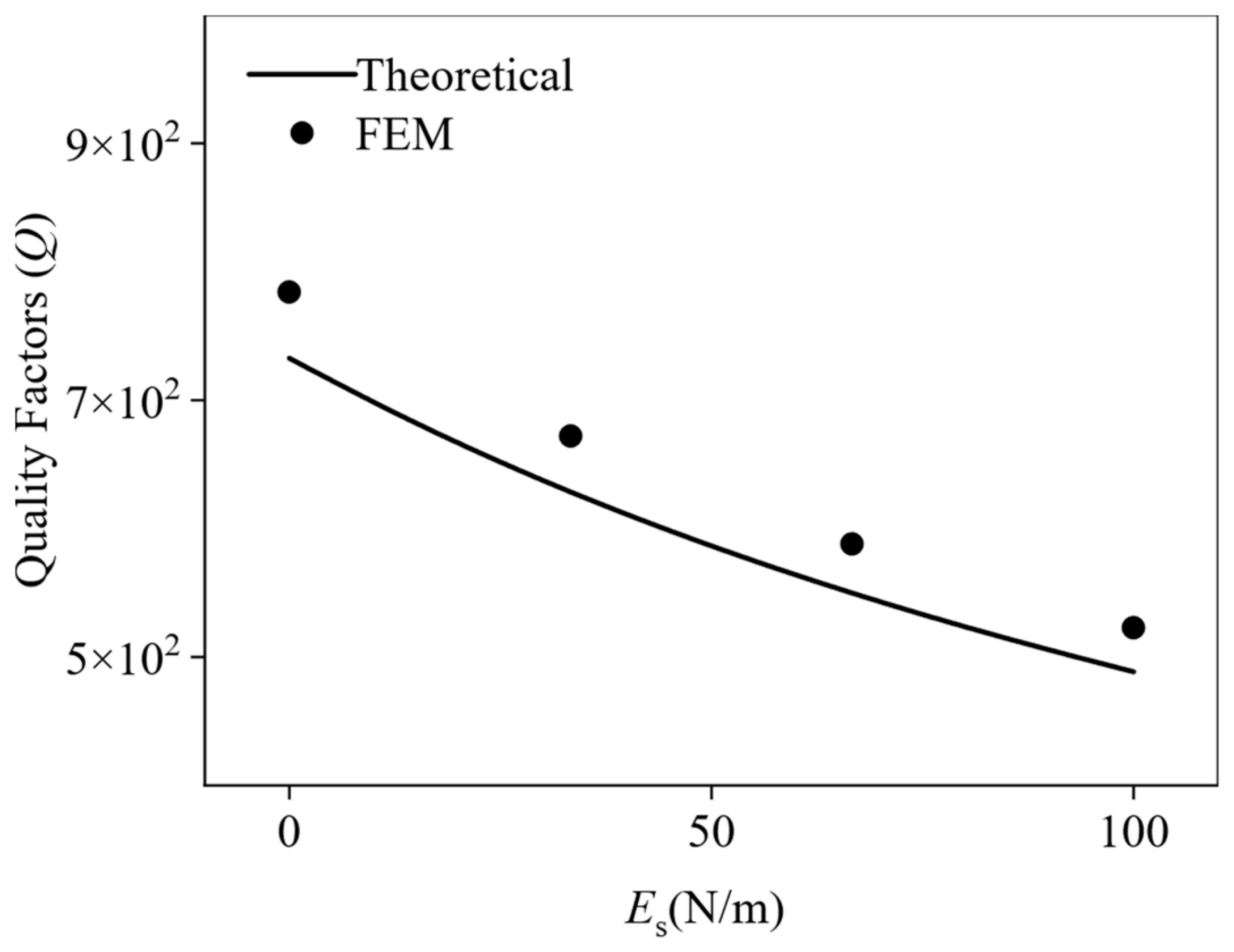
Effect of surface elastic modulus $E_{s}$ on support loss (mean relative error between theoretical and finite element results: 5.1%).

As shown in Figure 6, the surface elastic modulus considerably affects the quality factor of the double-clamped micro-/nano-beam resonator. Within the investigated parameter range, introducing the surface elastic modulus while neglecting the initial surface stress results in a monotonic decrease in the quality factor, indicating increased support loss. This behavior arises because the surface elastic modulus modifies the natural frequency, effective bending stiffness, and local mechanical response near the support regions.

Mechanistically, the surface elastic modulus increases the constraint imposed by the surface layer on beam deformation, leading to a higher effective bending stiffness during vibration. This modifies the modal response and shear-force distribution at the clamped ends. The resulting increase in natural frequency further strengthens the dynamic excitation transmitted from the support regions to the supports, thereby enhancing elastic-wave radiation and vibrational energy dissipation. Because support loss is governed by dynamic energy transfer from the resonator to the supports, a larger surface elastic modulus increases the transmitted stress disturbance and reduces the quality factor.

### 4.2. Effect of Initial Surface Stress

**Figure 7:**
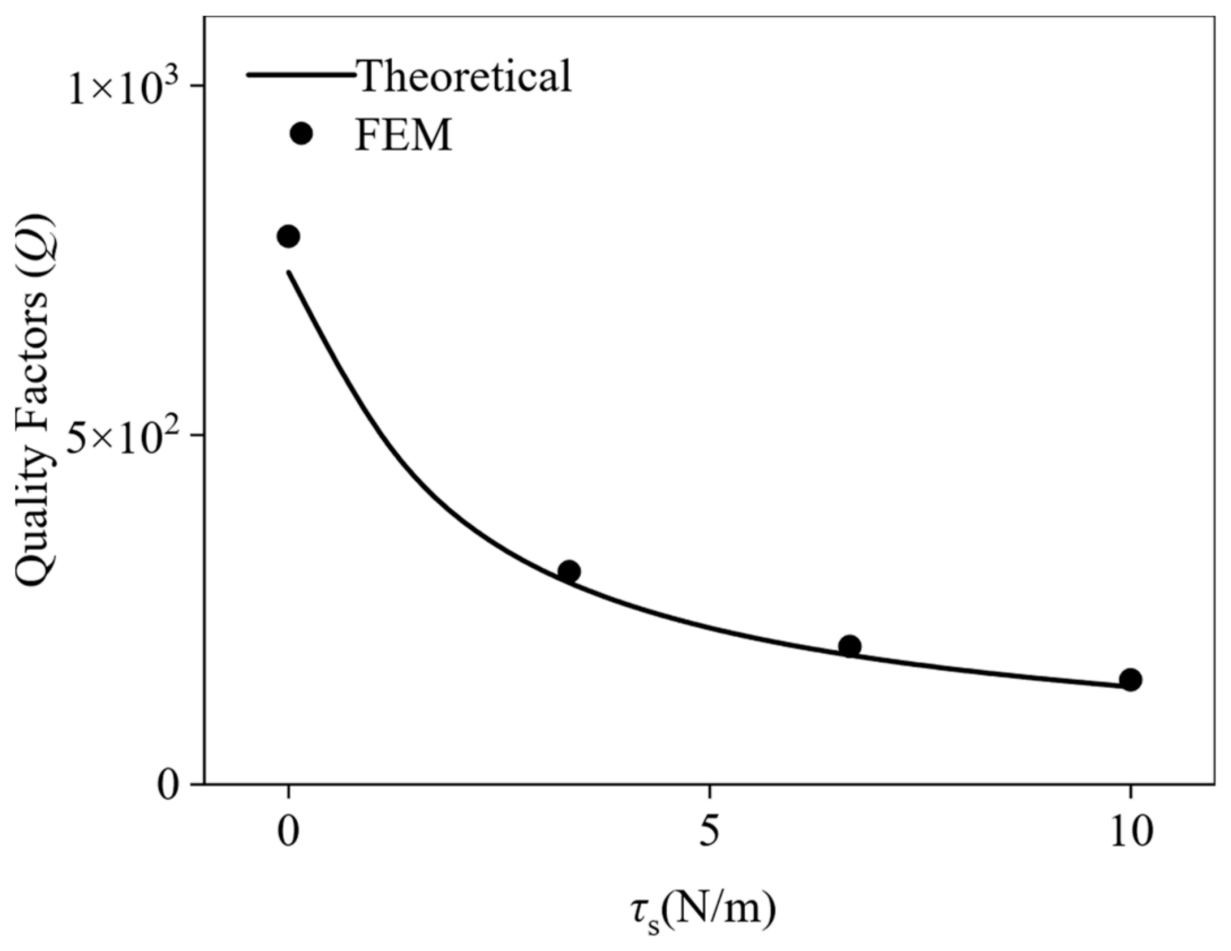
Effect of initial surface stress $\tau_{s}$ on support loss (mean relative error between theoretical and finite element results: 6.8%).

As shown in Figure 7, the initial surface stress considerably affects the quality factor of the double-clamped micro-/nano-beam resonator. Within the investigated range, when only the initial surface stress is considered and the surface elastic modulus is neglected, the quality factor decreases gradually with increasing initial surface stress, indicating enhanced support loss.

Mechanistically, increasing the initial surface stress enhances the local constraint response of the resonator during vibration and modifies the shear force at the clamped ends and associated interfacial stress levels. These changes increase the dynamic excitation of the supports, resulting in stronger elastic-wave radiation and greater support loss. Further, initial surface stress influences the local mechanical field near the support regions by altering the prestress level and modal response. Although the mechanistic pathways of the initial surface stress and surface elastic modulus differ, both change the dynamic load transmitted through the support regions, thereby increasing support loss and reducing the quality factor. These results indicate that initial surface stress is a key surface parameter in the support-loss analysis of micro-/nano-resonators, particularly at high stress levels.

### 4.3. Effect of Structural Characteristic Dimension

**Figure 8:**
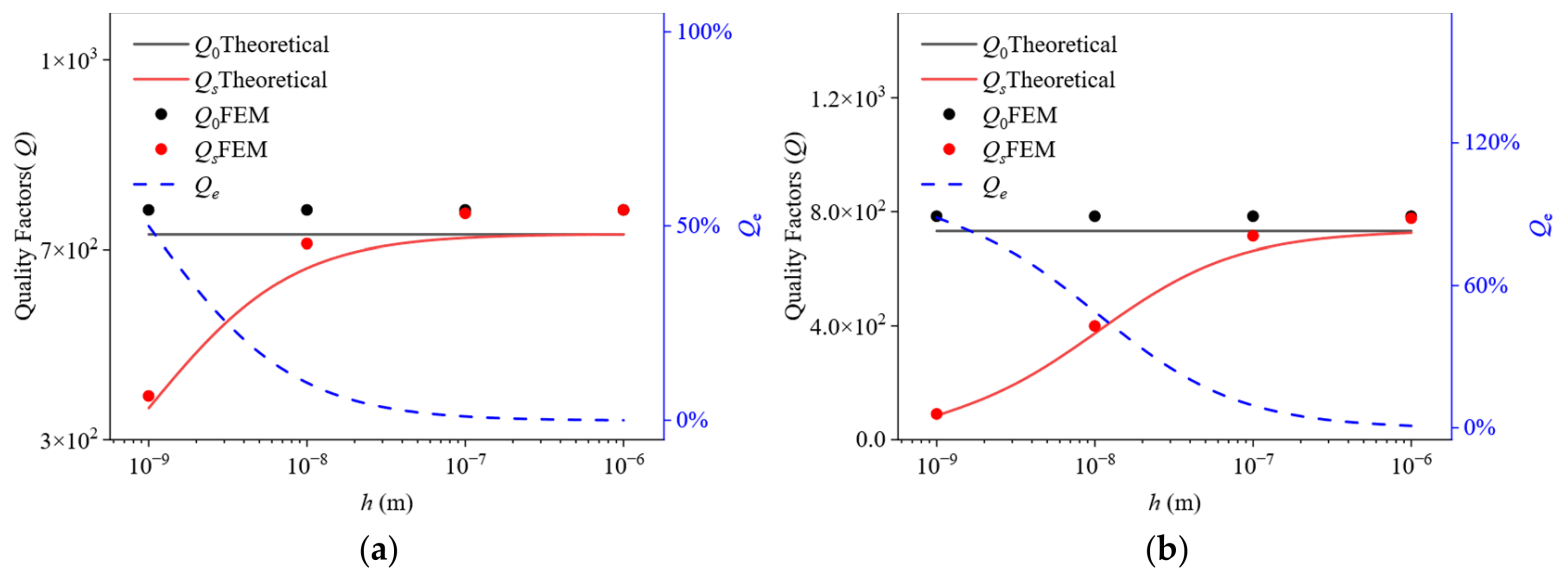
Influence of surface effects on support loss at different scales: (**a**) effect of surface elastic modulus only; (**b**) effect of initial surface stress only (mean relative errors between theoretical and finite element results: 6.4% and 7.1%, respectively).

When the dimensionless geometric parameters are fixed, the characteristic structural dimension governs the intensity of surface effects. As shown in Figure 8, the difference between the support loss predicted with and without surface effects gradually decreases as the characteristic dimension increases, indicating that surface effects become less pronounced at larger scales. Conversely, reducing the characteristic dimension enhances surface-effect contribution owing to the increased surface-area-to-volume ratio of micro-/nano-structures.

At larger scales, bulk material behavior dominates, while the contributions of surface layer stiffness and surface-induced stress to the overall mechanical response remain limited. Consequently, surface effects have minimal influence on the stress distribution near the support regions and associated elastic-wave radiation. As the characteristic dimension decreases, surface contributions become considerable, thereby amplifying the effects of the surface elastic modulus and initial surface stress on the effective stiffness, modal frequency, and support interface loads. Hence, support loss becomes more sensitive to surface parameters. The characteristic structural dimension determines the relative importance of surface effects in support-loss analysis.

### 4.4. Effect of Dimensionless Geometric Parameters

**Figure 9:**
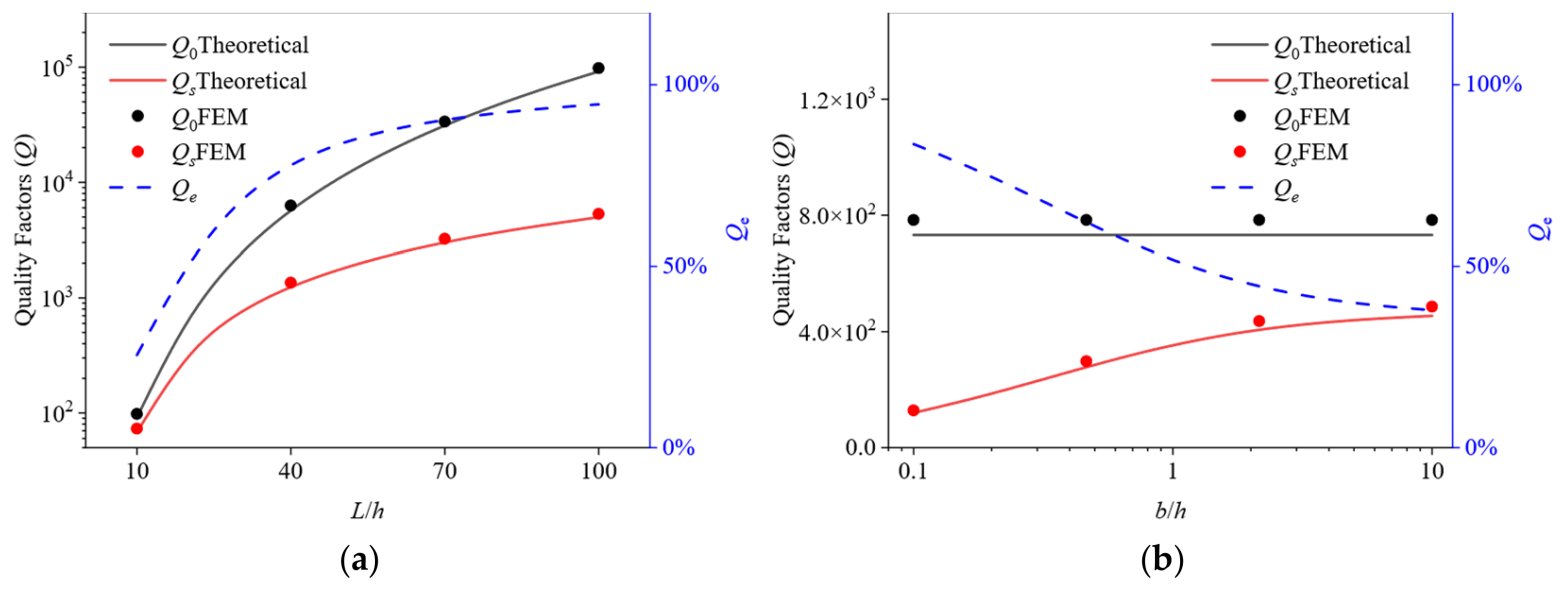
Influence of resonator geometric aspect ratios on surface-effect modulation of support loss: (**a**) effect of the resonator length-to-thickness ratio; (**b**) effect of the resonator width-to-thickness ratio (considering both surface elastic modulus and initial surface stress; mean relative errors between theoretical and finite element results: 4.7% and 5.6%, respectively).

In addition to the characteristic structural dimension, dimensionless geometric parameters equally influence the contribution of surface effects to support loss. As shown in Figure 9, variations in the length-to-thickness and width-to-thickness ratios modify the extent to which surface effects contribute to the overall support loss. Increasing the length-to-thickness ratio enhances the influence of surface effects.

As L/h increases, the beam becomes more slender, bending deformation more dominant, and stress transfer near the support regions more sensitive to the surface-layer stiffness and initial surface stress. Consequently, the influence of surface parameters on support loss is enhanced. The variation in the width-to-thickness ratio reflects the role of cross-sectional geometry in distributing surface contributions. For the in-plane vibration in the *xz*-plane herein, surface effects are governed by the side surface contribution associated with the thickness direction. Hence, when b/h decreases, the influence of surface parameters on the equivalent mechanical properties of the resonator and stress state near the support regions becomes more pronounced, thereby enhancing support loss. These results indicate that surface effects are coupled with the geometric configuration rather than acting as independent corrections. For resonators with a similar material and size scale, variations in the dimensionless geometric parameters can result in considerable changes in the quality factor. Hence, the design of high-quality-factor micro-/nano-resonators must account for both geometric and surface parameters in a coordinated manner.

## 5. Conclusions

Based on Euler–Bernoulli beam theory and Gurtin–Murdoch surface elasticity theory, a support-loss model for double-clamped micro-/nano-beam resonators is developed by considering surface effects. A 3D finite element model is developed to validate the theoretical results. The effects of surface parameters, characteristic structural dimensions, and dimensionless geometric parameters on support loss and the quality factor are systematically investigated. The main conclusions are as follows:

1. Both the surface elastic modulus and initial surface stress increase support loss and reduce the quality factor. Although their underlying mechanisms differ, both enhance vibrational energy dissipation by modifying the effective mechanical response of the resonator and local dynamic loads in the support region.
2. The characteristic structural dimension considerably influences the role of surface effects. For fixed dimensionless geometric parameters, the influence of surface effects on support loss and the quality factor becomes more pronounced as the characteristic dimension decreases and less pronounced as it increases.
3. Dimensionless geometric parameters regulate the influence of surface effects on support loss. Variations in the length-to-thickness and width-to-thickness ratios modify the surface contribution of the cross-section and mechanical response near the support regions, thereby altering the contribution of surface effects on support loss.

In summary, surface effects contribute considerably to the support loss of double-clamped micro-/nano-beam resonators, and their contribution is jointly governed by structural scale and dimensionless geometric parameters. It should be noted that the present investigation is theoretical and numerical in nature. In experimental characterizations of micro-/nano-resonators, the measured overall quality factor typically reflects the concurrent action of multiple coupled energy dissipation mechanisms (e.g., thermoelastic damping, air damping, surface damping, and support loss), which presents substantial technical challenges in directly isolating the specific contribution of surface effects to support loss. Within this context, the theoretical and numerical framework developed herein provides a viable means to decouple these loss channels and systematically evaluate the surface-dependent radiation mechanisms from a mechanics perspective. These findings provide a theoretical basis for support-loss analysis considering surface effects and offer insights for designing high-quality-factor micro-/nano-resonators.

## Figures and Tables

**Table 1:** Default values and variation ranges of the parameters.

Parameter	Description	Default Value	Variation Range
*h*	Resonator thickness	10 nm	1–1000 nm
*L/h*	Length-to-thickness ratio	20	10–100
*b/h*	Width-to-thickness ratio	1	0.6–10
$E_{b}$	Bulk elastic modulus	160 GPa	-
ρ	Density	2330 kg/m^3^	-
ν	Poisson’s ratio	0.28	-
$E_{s}$	Surface elastic modulus	20 N/m [27,46]	0–100 N/m
$\tau_{s}$	Initial surface stress	2 N/m [47]	0–10 N/m

## Data Availability

The original contributions presented in this study are included in the article. Further inquiries can be directed to the corresponding author.

## References

[1] Ekinci K.L., Roukes M.L. (2005). Nanoelectromechanical Systems. Rev. Sci. Instrum..

[2] Wei L., Kuai X., Bao Y., Wei J., Yang L., Song P., Zhang M., Yang F., Wang X. (2021). The Recent Progress of MEMS/NEMS Resonators. Micromachines.

[3] Yazdi N., Ayazi F., Najafi K. (1998). Micromachined Inertial Sensors. Proc. IEEE.

[4] Hanay M.S., Kelber S., Naik A.K., Chi D., Hentz S., Bullard E.C., Colinet E., Duraffourg L., Roukes M.L. (2012). Single-Protein Nanomechanical Mass Spectrometry in Real Time. Nat. Nanotechnol..

[5] Nguyen C.T.-C. (2007). MEMS Technology for Timing and Frequency Control. IEEE Trans. Ultrason. Ferroelectr. Freq. Control.

[6] Feng T., Yuan Q., Yu D., Wu B., Wang H. (2022). Concepts and Key Technologies of Microelectromechanical Systems Resonators. Micromachines.

[7] Abdolvand R., Bahreyni B., Lee J.E.-Y., Nabki F. (2016). Micromachined Resonators: A Review. Micromachines.

[8] Arlett J.L., Myers E.B., Roukes M.L. (2011). Comparative Advantages of Mechanical Biosensors. Nat. Nanotechnol..

[9] Ferrari P.F., Kim S., van der Zande A.M. (2023). Nanoelectromechanical Systems from Two-Dimensional Materials. Appl. Phys. Rev..

[10] Wang Z., Shi N., Zhang Y., Zheng N., Li H., Jiao Y., Cheng J., Wang Y., Zhang X., Chen Y. (2023). Conformal In-Ear Bioelectronics for Visual and Auditory Brain-Computer Interfaces. Nat. Commun..

[11] Xu J., Xi J., Wang C., Kraft M., Liu H., Martins R.P., Mak P.-I., Wang Y. (2025). A Multi-Functional MEMS Resonator for Simultaneously Dual-Mode Physical Sensing and ppb-Level Timing. Microsyst. Nanoeng..

[12] Miller J.M.L., Ansari A., Heinz D.B., Chen Y., Flader I.B., Shin D.D., Villanueva L.G., Kenny T.W. (2018). Effective Quality Factor Tuning Mechanisms in Micromechanical Resonators. Appl. Phys. Rev..

[13] Arash B., Jiang J., Rabczuk T. (2015). A Review on Nanomechanical Resonators and Their Applications in Sensors and Molecular Transportation. Appl. Phys. Rev..

[14] Chouvion B., McWilliam S., Popov A.A., Fox C.H.J. (2012). Review and Comparison of Different Support Loss Models for Micro-Electro-Mechanical Systems Resonators Undergoing in-Plane Vibration. Proc. Inst. Mech. Eng. Part C J. Mech. Eng. Sci..

[15] Hao Z., Erbil A., Ayazi F. (2003). An Analytical Model for Support Loss in Micromachined Beam Resonators with In-Plane Flexural Vibrations. Sens. Actuators A.

[16] Hao Z., Xu Y. (2009). Vibration Displacement on Substrate Due to Time-Harmonic Stress Sources from a Micromechanical Resonator. J. Sound Vib..

[17] Cross M.C., Lifshitz R. (2001). Elastic Wave Transmission at an Abrupt Junction in a Thin Plate with Application to Heat Transport and Vibrations in Mesoscopic Systems. Phys. Rev. B.

[18] Judge J.A., Photiadis D.M., Vignola J.F., Houston B.H., Jarzynski J. (2007). Attachment Loss of Micromechanical and Nanomechanical Resonators in the Limits of Thick and Thin Support Structures. J. Appl. Phys..

[19] Wilson-Rae I. (2008). Intrinsic Dissipation in Nanomechanical Resonators Due to Phonon Tunneling. Phys. Rev. B.

[20] Hao Z., Ayazi F. (2007). Support Loss in the Radial Bulk-Mode Vibrations of Center-Supported Micromechanical Disk Resonators. Sens. Actuators A.

[21] Ma C., Wei A., Shi K., Zhao Y., Yang W., Chen S., Guo F. (2023). The Role of Axial Pre-Tension in Reducing Energy Dissipation of Micro/Nano-Mechanical Resonators. Eur. J. Mech. A Solids.

[22] Ma C., Wang J., Shi K., Kong Z., Yang W., Chen S., Guo F. (2024). Nonlinear Support Loss in Micro/Nano Beam Resonators Induced by Geometric Nonlinearity. Acta Mech. Sin..

[23] Xiao Y., Zhu K., Han J., Liu S., Wu G. (2024). Q-Enhancement of Piezoelectric Micro-Oven-Controlled MEMS Resonators Using Honeycomb Lattice Phononic Crystals. Chip.

[24] Xiao S., Peng Z., Wu H., Yao Y., Chen S. (2023). Surface Effect in Nano-Scale Fretting Contact Problems. ASME J. Appl. Mech..

[25] Neffati D., Kulkarni Y. (2022). Homogenization of Surface Energy and Elasticity for Highly Rough Surfaces. ASME J. Appl. Mech..

[26] Gurtin M.E., Murdoch A.I. (1975). A Continuum Theory of Elastic Material Surfaces. Arch. Ration. Mech. Anal..

[27] Miller R.E., Shenoy V.B. (2000). Size-Dependent Elastic Properties of Nanosized Structural Elements. Nanotechnology.

[28] Jing G., Duan H., Sun X., Zhang Z., Xu J., Li Y., Wang J., Yu D. (2006). Surface Effects on Elastic Properties of Silver Nanowires: Contact Atomic-Force Microscopy. Phys. Rev. B.

[29] Cuenot S., Frétigny C., Demoustier-Champagne S., Nysten B. (2004). Surface Tension Effect on the Mechanical Properties of Nanomaterials Measured by Atomic Force Microscopy. Phys. Rev. B.

[30] Chen C., Shi Y., Zhang Y., Zhu J., Yan Y. (2006). Size Dependence of Young’s Modulus in ZnO Nanowires. Phys. Rev. Lett..

[31] Roy A., Mead J., Wang S., Huang H. (2017). Effects of Surface Defects on the Mechanical Properties of ZnO Nanowires. Sci. Rep..

[32] Guo J., Zhao Y. (2007). The Size-Dependent Bending Elastic Properties of Nanobeams with Surface Effects. Nanotechnology.

[33] He J., Lilley C.M. (2008). Surface Effect on the Elastic Behavior of Static Bending Nanowires. Nano Lett..

[34] Ansari R., Gholami R., Faghih Shojaei M., Mohammadi V., Sahmani S. (2013). Surface Stress Effect on the Vibrational Response of Circular Nanoplates with Various Edge Supports. ASME J. Appl. Mech..

[35] Park H.S. (2008). Surface Stress Effects on the Resonant Properties of Silicon Nanowires. J. Appl. Phys..

[36] Yao Y., Chen S. (2015). Surface Effect on Resonant Properties of Nanowires Predicted by an Elastic Theory for Nanomaterials. J. Appl. Phys..

[37] Pyziak L., Wojnarowska W., Wanic M., Oliwa R., Więcek T., Chotorlishvili L. (2026). Modal Origin of the Temperature-Dependent DMA Phase Shift in Polymer Beams. Acta Mech. Autom..

[38] Liu C., Rajapakse R.K.N.D., Phani A.S. (2011). Finite Element Modeling of Beams with Surface Energy Effects. ASME J. Appl. Mech..

[39] Chen S., Liu J., Guo F. (2017). Evaluation of Support Loss in Micro-Beam Resonators: A Revisit. J. Sound Vib..

[40] Frangi A., Bugada A., Martello M., Savadkoohi P.T. (2013). Validation of PML-Based Models for the Evaluation of Anchor Dissipation in MEMS Resonators. Eur. J. Mech. A Solids.

[41] Steeneken P.G., Ruigrok J.J.M., Kang S., van Beek J.T.M., Bontemps J., Koning J.J. Parameter Extraction and Support-Loss in MEMS Resonators. Proceedings of the COMSOL Users Conference 2007.

[42] Hu K., Zhang W., Zhong Z., Peng Z., Meng G. (2014). Effect of Surface Layer Thickness on Buckling and Vibration of Nonlocal Nanowires. Phys. Lett. A.

[43] Hu K., Zhang W., Peng Z., Meng G. (2016). Transverse Vibrations of Mixed-Mode Cracked Nanobeams with Surface Effect. ASME J. Vib. Acoust..

[44] Zhang W., Hu K., Yang B., Peng Z., Meng G. (2016). Effects of Surface Relaxation and Reconstruction on the Vibration Characteristics of Nanobeams. J. Phys. D Appl. Phys..

[45] Chen S., Yang S., Ma C., Liu Q., Jiao W., Chen Y., Zhang Y. (2026). Mechanics Analysis of Support Loss Mechanisms in Flexible MEMS Resonators. J. Sound Vib..

[46] Dingreville R., Qu J., Cherkaoui M. (2005). Surface Free Energy and Its Effect on the Elastic Behavior of Nano-Sized Particles, Wires and Films. J. Mech. Phys. Solids.

[47] Ibach H. (1997). The Role of Surface Stress in Reconstruction, Epitaxial Growth and Stabilization of Mesoscopic Structures. Surf. Sci. Rep..

